# Research Wranglers: Initiatives to Improve Reproducibility of Study Findings

**DOI:** 10.1289/ehp.122-A188

**Published:** 2014-07-01

**Authors:** Charles W. Schmidt

**Affiliations:** Charles W. Schmidt, MS, an award-winning science writer from Portland, ME, has written for *Discover Magazine*, *Science*, and *Nature Medicine*.

Advances in science depend on researchers being able to reproduce the findings of their peers, thus providing a solid platform from which to move forward with new lines of scientific inquiry. Yet for a variety of reasons, irreproducibility appears to be a growing problem in experimental research. Now funding agencies and research journals are crafting guidelines to ensure that published studies are well designed, well reported, and better able to generate reproducible results.

**Figure:**
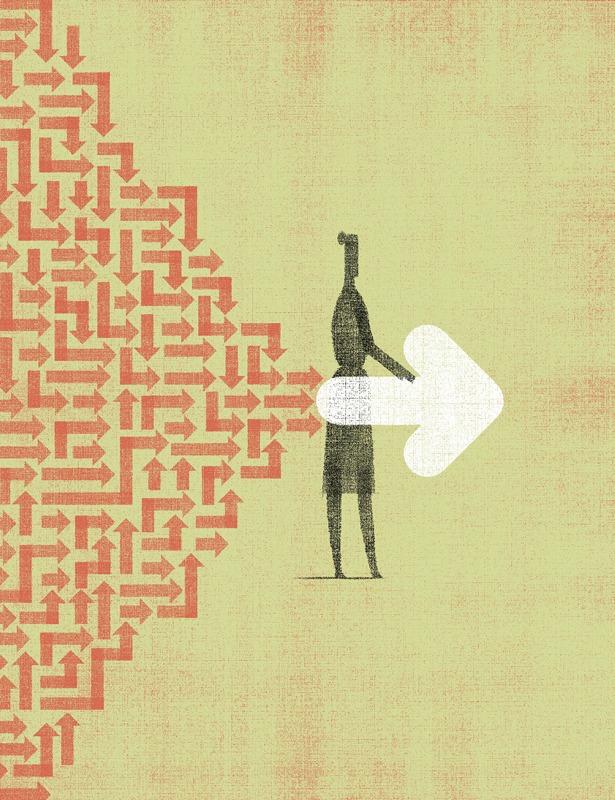
© Jim Frazier

Media reports have singled out a number of egregious cases of irreproducibility, including reports published in *Nature* earlier this year indicating that adult stem cells would become pluripotent if submerged in a mild acid bath.^1^^,^^2^ The findings were widely criticized after scientists were unable to reproduce the results in their own laboratories.^3^ Subsequently, the first author agreed to retract the reports.^4^ In another widely publicized example, the biopharmaceutical company Amgen claimed it could reproduce just 6 of 53 studies that were considered landmarks in basic cancer research, despite close cooperation with the original scientists to make sure the same experimental protocols were used.^5^

Experts blame the irreproducibility problem on a number of factors, including inadequate reporting of the methods used in published research studies. Mounting evidence links poor methods descriptions with overstated findings,^6^^,^^7^^,^^8^^,^^9^^,^^10^ but in other cases faulty conclusions can be attributed to poor study design. In particular, inadequate randomization and blinding can introduce biases that render a study incapable of accurately testing its thesis.^11^

Irreproducibility is especially problematic if it plagues findings that lead to human clinical trials or to regulations and policies that could affect public health. One such instance was documented by researchers at the National Institute of Neurological Disorders and Stroke (NINDS), who discovered that patients with amyotrophic lateral sclerosis had been enrolled in a clinical trial founded on inadequate preclinical data.

According to NINDS director Story Landis, the patients weren’t doing nearly as well as hoped on the test treatment, a broad-spectrum antibiotic called minocycline. When NINDS researchers took a closer look at the preclinical studies upon which the trial was predicated, they found the authors had not reported whether the studies were randomized or blinded. Furthermore, the work was done using small numbers of animals. “That was a wake-up call,” Landis says. “Human clinical trials need to be based on solid preclinical findings.”

## New Initiatives

At a fundamental level, “reproducibility” refers to the ability of scientists to generate results comparable to those reported in prior studies. Thus, reproducibility differs from replication, or the generation of precisely the same results using the same experimental methods as the original investigators. The terms are often used interchangeably, but NINDS program director Shai Silberberg argues that replication is more of an ideal than a practical goal.

“That’s because in practice, there will always be variables that we can’t control, making true replication impossible,” Silberberg says. For instance, researchers can’t use the same animals twice, he explains, and the use of a different set of animals—even of the same age, sex, and strain—introduces variability into experimental conditions.

In January 2014 the leadership of the National Institutes of Health (NIH) announced new initiatives to confront the problem of irreproducible research.^12^ NIH principal deputy director Lawrence Tabak says the inability to reproduce peer-reviewed results can stand in the way of scientific progress. “We need to build on a firm basis of prior findings to make advances in research,” he says. “And that’s true of all science, not just what we do at the NIH.”

Described in a *Nature* commentary coauthored by Tabak and NIH director Frances Collins,^12^ the initiatives include training in experimental design and implementation of checklists to ensure that funding applicants sufficiently address randomization, blinding, and appropriate statistical methods. The NIH is also pursuing development of a Data Discovery Index^13^ to provide access to unpublished primary data. By providing such access, researchers may be able to discover cases where irreproducibility stems from errors in data analysis or use of inappropriate analytic methods. Finally, a pilot program called PubMed Commons^14^ offers researchers an open forum to discuss articles indexed in PubMed.

On the journal front, Nature Publishing Group, *Science*, and *Science Translational Medicine* have each announced their own measures to address reproducibility. “We’re in a position to be part of the solution,” says *Science* editor-in-chief Marcia McNutt. According to McNutt, funders can address reproducibility before experiments begin, while publishers can increase transparency into how the experiments were carried out by encouraging authors to describe laboratory and statistical methods with sufficient detail. That way, other scientists can determine their own level of confidence in the results and confirm reported results.

“We’re asking authors, ‘Did you run enough samples? Were you blind as to the makeup of treatment groups and controls? Did you have a pre-experimental plan for dealing with outliers, or did you change the rules on the fly?’” McNutt explains. Reviewers and editors at *Science* are now flagging papers that demonstrate exemplary transparency with the aim of developing additional reproducibility guidelines later this year.^15^

## Preclinical Studies under Scrutiny

Both the NIH and publisher initiatives are focusing at the outset on preclinical experimental research. That’s in part because human interventions are often predicated on preclinical animal data, and also because rigorous efforts to limit bias and bolster confidence in scientific findings are routinely applied in clinical trials with human subjects.^12^

McNutt adds that concerns over preclinical reproducibility also led to consensus community standards derived from a June 2012 workshop that was convened by the NINDS and attended by about 50 stakeholders from academia, publishing, advocacy groups, funding agencies, and the pharmaceutical industry.^16^ Attendants broadly agreed that poor methods reporting and poor experimental design often go hand-in-hand, and that recommendations to address the problem were needed.^16^

According to Silberberg, just because methods aren’t adequately described in a paper doesn’t mean the experiment wasn’t well conducted. But investigators need to pay better attention to experimental design, Landis says, as well as to the statistical methods they use, especially for complex data sets emerging from high-throughput research.

Guidelines addressing research reporting were already in existence at the time of the NINDS workshop, notably the ARRIVE (Animal Research: Reporting *in Vivo* Experiments) guidelines developed by British researchers and published in 2010.^17^ The ARRIVE guidelines encourage better methods reporting in peer-reviewed papers, and many research funding bodies and publishers (including *EHP*) have adopted them. But according to Silberberg, the guidelines are comprehensive and detailed to the point of unwieldiness. “There are really important items in it and others that are not so important,” he says. “If you dictate how to write your abstract and your title, you lose your audience.”

**Figure:**
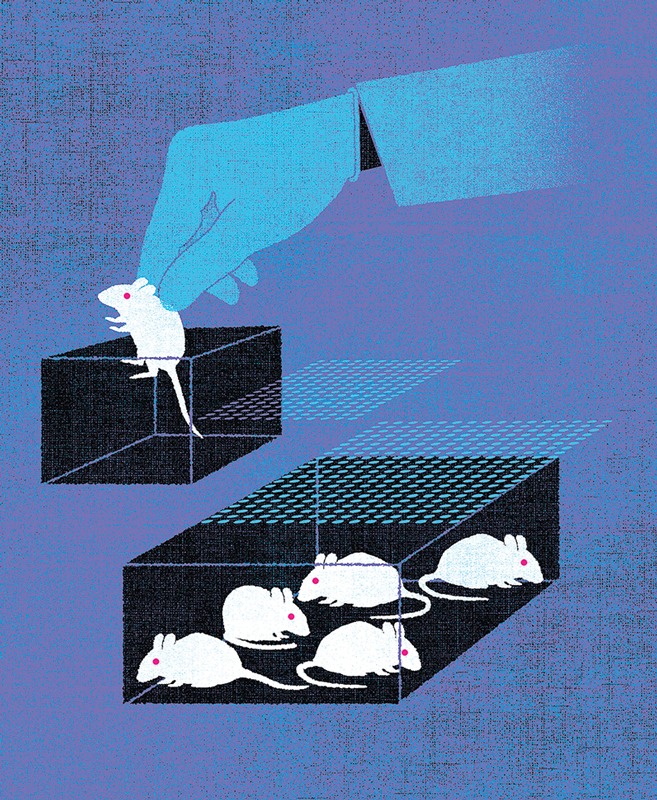
NINDS’s Streamlined Recommendations for Reporting Standards **Randomization** Animals should be assigned randomly to various experimental groups and the method of randomization reported.Data should be collected and processed randomly or appropriately blocked. **Blinding** Allocation concealment: the investigator should be unaware of the group to which the next animal taken from a cage will be allocated.Blinded conduct of the experiment: animal caretakers and investigators conducting the experiments should be blinded to the allocation sequence.Blinded assessment of outcome: investigators assessing, measuring, or quantifying experimental outcomes should be blinded to the intervention.

**Figure:**
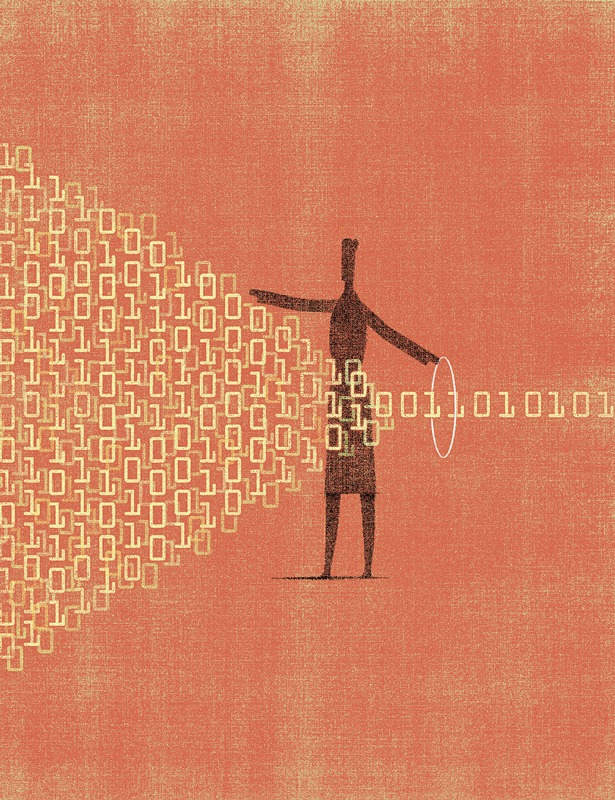
**Sample-size estimation** An appropriate sample size should be computed when the study is being designed and the statistical method of computation reported.Statistical methods that take into account multiple evaluations of the data should be used when an interim evaluation is carried out. **Data handling** Rules for stopping data collection should be defined in advance.Criteria for inclusion and exclusion of data should be established prospectively.Criteria for defining and handling outliers should be decided when the experiment is being designed, and any data removed before analysis should be reported.The primary end point should be prospectively selected. If multiple end points are to be assessed, then appropriate statistical corrections should be applied.Investigators should report on data missing because of attrition or exclusion.Pseudoreplication issues need to be considered during study design and analysis.Investigators should report how often a particular experiment was performed and whether results were substantiated by repetition under a range of conditions. Recommendations adapted from Landis et al. (2012).^16^ Both images: © Jim Frazier

Attendees at the NINDS workshop came up with a more limited set of recommendations that were published the following October in *Nature*.^16^ These recommendations call on researchers to, at a minimum, report how sample sizes were determined, whether and how animals were randomized in the study, whether the investigators were blind to treatment, and how the data were handled.

Each of these factors is crucial to good experimental design. By allocating animals randomly to treatment or control groups and then blinding themselves to outcome, for instance, investigators limit the potential for introducing confounders that influence study results, says Jim Berger, a professor of statistics at Duke University. Likewise, appropriate sample sizes are necessary to ensure that findings are statistically viable.

## The Value of Checklists

In the wake of the NINDS workshop, Nature Publishing Group announced a reproducibility initiative in April 2013.^18^ With it, *Nature* lifted length restrictions for methods reporting in online supplements. In addition, both authors and reviewers now have to complete checklists for experimental design. Finally, the journal hired statisticians to help with review, and it provided a mechanism by which the public can access raw data used to generate published tables and figures. The NINDS recommendations are also cited by McNutt as the inspiration behind *Science*’s new reproducibility initiative.^15^

The NIH initiatives on reproducibility remain a work in progress, Tabak emphasizes, but they put training at the forefront. New training modules geared toward intramural trainees will cover basic issues in experimental design, and the NIH will produce short films on key topics such as randomization, blinding, and gender differences in animal response, which Silberberg says will be made available both within and outside the institutes.

Apart from the teaching modules, the NIH is considering how to incorporate checklists into the review of grant applications. The checklists are expected to cover standard experimental design features (i.e., randomization, blinding, and statistics), but Silberberg says there’s also a deliberate attempt among those developing the initiatives to “do no harm,” or in other words, to avoid stifling creativity.

Silberberg carefully distinguishes between hypothesis-testing research (which has strict methodological requirements) and hypothesis-generating research (which doesn’t). “You shouldn’t have to adhere to strict rules when you’re doing exploratory studies,” he says. “We don’t want our reviewers to be too narrow-minded if the research has exciting potential.”

A number of NIH centers and institutes are now developing and testing checklists based on a variety of research questions.^19^ Informed by those pilots, Tabak says, NIH leaders will decide later this year which to adopt agency-wide, which should remain specific to particular institutes and centers, and which to drop.

## NIEHS Approach

The NIEHS, meanwhile, has developed a framework for systematic review of published research, which it uses in its assessments of potential hazards. These assessments are carried out by reviewers in the Office of Health Assessment and Translation (OHAT) within the NIEHS National Toxicology Program (NTP). OHAT reviewers conduct technical assessments to identify the potential for harm caused by substances in the environment. Basing these assessments on poor-quality studies can lead to faulty conclusions and thus to policies that go either too far or not far enough in terms of minimizing risk.

Beginning in 2011 OHAT began exploring systematic review as a means of weeding out substandard studies, particularly for health effects other than cancer. But while the method was well established in clinical medicine, it wasn’t adapted to decision-making in environmental health, which relies on data from more diverse sources, including epidemiology and animal toxicology studies, in addition to mechanistic studies with *in vitro* systems. OHAT therefore worked with technical experts on how to modify systematic review for its own purposes, publishing a draft seven-step framework in 2013.^20^

That framework has now been finalized^21^ and is being implemented. A reviewer may still reach an incorrect conclusion using systematic review, but there will be a transparent window into how the decision was reached. According to John Bucher, associate director of the NTP, the framework makes it possible to consistently aggregate a diversity of data. “We’re trying to create a trail of judgments on individual studies that can be followed to explain our confidence in the overall evidence,” he says.

Still, even the best-conducted studies can’t control for every variable that might affect reproducibility. In some cases, the experimental reagents themselves are highly vulnerable to change. Epithelial cell lines, for instance, are exquisitely sensitive to slight shifts in their microenvironment, and even highly skilled scientists can unknowingly introduce changes that affect experimental outcomes, wrote Mina Bisell, a cancer researcher at Lawrence Berkeley National Laboratory, in a 2013 commentary on reproducibility.^22^

**Figure:**
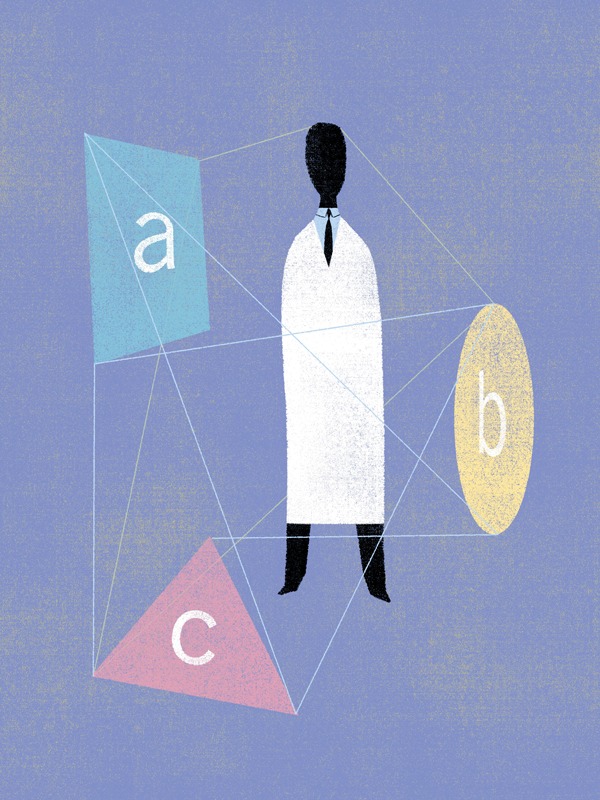
OHAT’s Seven-Step Systematic Review Process **Step 1**: Formulate problem, and develop protocol. **Step 2**: Search for and select studies for inclusion. **Step 3**: Extract data from studies. **Step 4**: Assess the quality or risk of bias of individual studies. **Step 5**: Rate the confidence in the body of evidence: Initial confidence set by key features of study design for each outcome.Downgrade or upgrade confidence rating as needed.Combine confidence conclusions for all study types and multiple outcomes. **Step 6**: Translate the confidence ratings into a level of evidence for health effects. **Step 7**: Integrate the evidence to develop hazard identification conclusions. Steps adapted from Rooney et al. (2014).^21^ Image: © Jim Frazier

Rick Woychik, deputy director of the NIEHS and prior president of The Jackson Laboratory, points out that in just 10–20 generations, mouse strains bred for specific research purposes can evolve genetic differences that may affect how they respond to environmental chemicals and pharmaceuticals. Moreover, he says, it is critically important for investigators to understand exactly which genetic background they are using because it can profoundly influence an experimental outcome.

“Some inbred mouse strains have high blood pressure, some don’t. Some are highly sensitive to drugs like acetaminophen, and some aren’t. The list of variable traits goes on and on,” Woychik explains. “Most notably, a knockout of a gene can have a different phenotype on different genetic backgrounds. So if the strain’s genetic background is not carefully controlled between two labs that are studying, for example, the same knockout of a gene, you can’t expect to get the same result.”

Bucher adds that the NIEHS has been monitoring genetic drift in its rodent colonies for 30 years. If needed, he says, it’s possible in some cases to re-establish genetically defined mouse lines from cryopreserved embryos. In addition, innovative new reference mouse panels known as the Collaborative Cross^23^ and Diversity Outbred^24^ are gaining momentum at the NIEHS. “These two reference panels more accurately reflect the phenotypic variability that exists within the human population than any single inbred strain,” Woychik says.

## No Silver Bullet

“There won’t be any silver bullet to solve the reproducibility problem,” says Paula Stephan, a professor at Georgia State University who in 2011 reported that some countries—notably China, Turkey, and South Korea—offer cash bonuses equal to as much as 7.5% of annual faculty salary to publish in top journals.^25^ She adds, “The burden on review has increased dramatically, and the quality of submitted papers is declining.”

But with instances of irreproducibility growing, “now is the time to raise awareness among all our stakeholders,” Tabak says. “We need to emphasize that the NIH can’t resolve this problem by itself. We need all our stakeholders—including journal editors, reviewers, and those in the university system—working together to try to address it.”
